# Telemonitoring Techniques for Lung Volume Measurement: Accuracy, Artifacts and Effort

**DOI:** 10.3389/fdgth.2020.559483

**Published:** 2020-09-17

**Authors:** Denise C. Mannée, Frans de Jongh, Hanneke van Helvoort

**Affiliations:** ^1^Pulmonary Department, Radboudumc, Nijmegen, Netherlands; ^2^Pulmonary Department, Medisch Spectrum Twente, Enschede, Netherlands

**Keywords:** Telemonitoring, Lung volumes, accuracy, artifacts, effort

## Abstract

Telemonitoring becomes more important in pulmonary research. It can be used to decrease the pressure on the health care system, to lower the costs of health care and to increase quality of life of patients. Previous studies show contradictory results regarding the effectiveness of telemonitoring. According to multiple researchers, inefficiency can be a result of poor study design, low data quality and usability issues. To counteract these issues, this review proves for an in-depth explanation of four (potential) telemonitoring systems in terms of work principle, accuracy, disturbing factors and usability. The evaluated systems are portable spirometry/breath-by-breath analyzers, respiratory inductance and magnetic plethysmography and electrical impedance tomography. These insights can be used to select the optimal technique for a specific purpose in future studies.

## Introduction

Home-based measurement of respiratory parameters has become a relevant topic in pulmonary research (1, 2). Multiple reviews have been dedicated to describe the effectiveness of telemonitoring for patients with pulmonary disease (3–6). Both Cruz et al. and Ambrosino et al. report a positive effect of telemonitoring on the disease, i.e., reduction of hospital admissions, mortality and health costs (5–7) and an improvement in quality of life for patients. Other researchers describe more inconsistent results (4, 8–11). Variations in results can be explained by the design of the study (4), usability issues (9), and the quality of data (11). To correctly interpret the data, it is important to acquire in depth knowledge on the functionality and accuracy of these techniques. Moreover, factors that potentially influence the quality of the data should be addressed. Lastly, it should be known how specific techniques are perceived by the patient and technician.

In multiple reviews the operation principle of various telemonitoring systems is described. Moreover, these reviews contain information on which parameters can be measured with these techniques (12–14). However, a profound explanation of various techniques integrated with information on accuracy, usability, and data quality is still lacking. In this review we elaborate on spirometry, respiratory inductance plethysmography (RIP), respiratory magnetic plethysmography (RMP), and electrical impedance tomography (EIT). The aim of this review is to describe the operation principles of these techniques, the calibration procedures, the accuracy of each device, the possible causes of inaccuracy. and the usability in perspective of the patient and technician. This review can be used by researchers to make a considered choice on the method of preference in their study involving telemonitoring and may result in better designed studies and increased data quality.

## Lung Volumes

**Figure 1:**
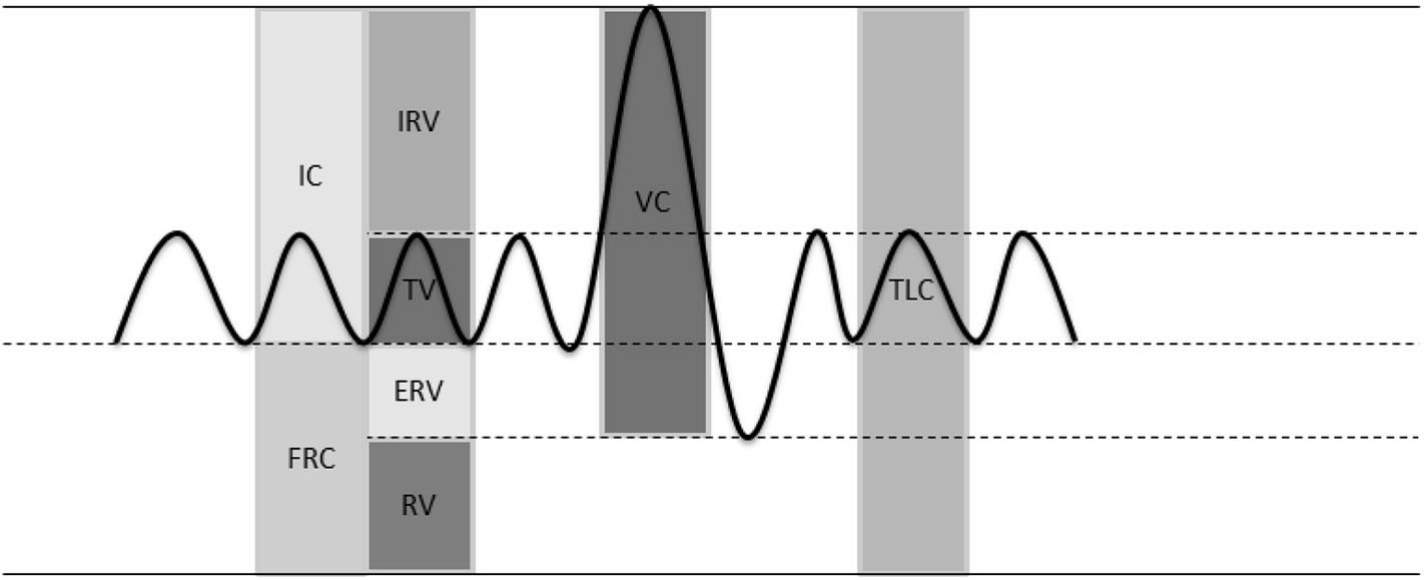
Lung volumes. Inspiratory capacity (IC), functional residual capacity (FRC), inspiratory reserve volume (IRV), tidal volume (TV), expiratory reserve volume (ERV), residual volume (RV), vital capacity (VC), total lung capacity (TLC).

Lung volumes can be classified by a set of variables (Figure 1) (15). These variables are divided into static and dynamic lung volumes. Dynamic lung volumes (i.e., inhaled and exhaled lung volumes) are useful for detecting, characterizing and quantifying the severity of pulmonary disease (16). TV, (F)VC, and forced expiratory volume in one second (FEV1) are considered dynamic volumes (15, 17, 18). FVC and FEV1 are also defined as functional lung volumes. At present, the most common method to measure these volumes is spirometry. However, the dynamic volumes are often not sufficient for diagnosis. Static lung volumes (RV, FRC or EELV, and TLC) might be complementary (19). Various methods are available for the measurement of static volumes, e.g., gas dilution methods, body plethysmography, nitrogen washout and radiographic imaging methods (19). All the stated measurement setups are used to measure lung volumes in a lung function lab and are not suitable for telemonitoring (15, 17–19).

Wearable/portable spirometers and breath-by-breath analyzers (BbB analyzer) were developed to be able to assess patients outside the lung function laboratory. In addition to these methods a diversity of devices has been developed to remotely monitor the respiratory parameters of a patient; respiratory plethysmography with use of inductive sensors (20), magnetometers (21) (RIP and RMP), and electrical (or electromagnetic) impedance tomography (EIT) (22). In which the latter, has not been investigated in telemonitoring setting, but has the potential for home monitoring (23, 24).

## Measurement Devices and Their Operation Principles

### Spirometry

Spirometers can be divided into two main groups, volume and flow measurement devices (25). Both methods can only measure dynamic volumes. Therefore, static volumes cannot be assessed with spirometers (19). Volume measurement devices (dry and wet spirometers) measure the exact volume displaced by the subject. In this method, the subject and the spirometer form a closed system. Consequently, any change in lung volume will give the exact opposite change in the volume of the spirometer. The hygiene of volume measurement devices is not easily regulated (16). Therefore, the most commonly used devices are flow measurement systems. In these systems, the subject is connected to a flow sensor with an air-tight connection by means of a face mask or mouth piece. The flow generated by the subject is integrated over time to obtain a volume. Flow can be measured with various sensors (25):

1. a pneumotachograph: flow is determined from a pressure drop measured over a known resistance, similar to Ohms law.
2. an ultrasonic sensor: flow is based on the Doppler effect or the Karman vortex effect.
3. a mass flow sensor: flow is derived from a decrease in resistance caused by cooling of heated wires in the system, caused by the breath of the subject.
4. a rotating vane system: flow is obtained from the number of rotations of the vane per unit of time.

**Figure 2:**
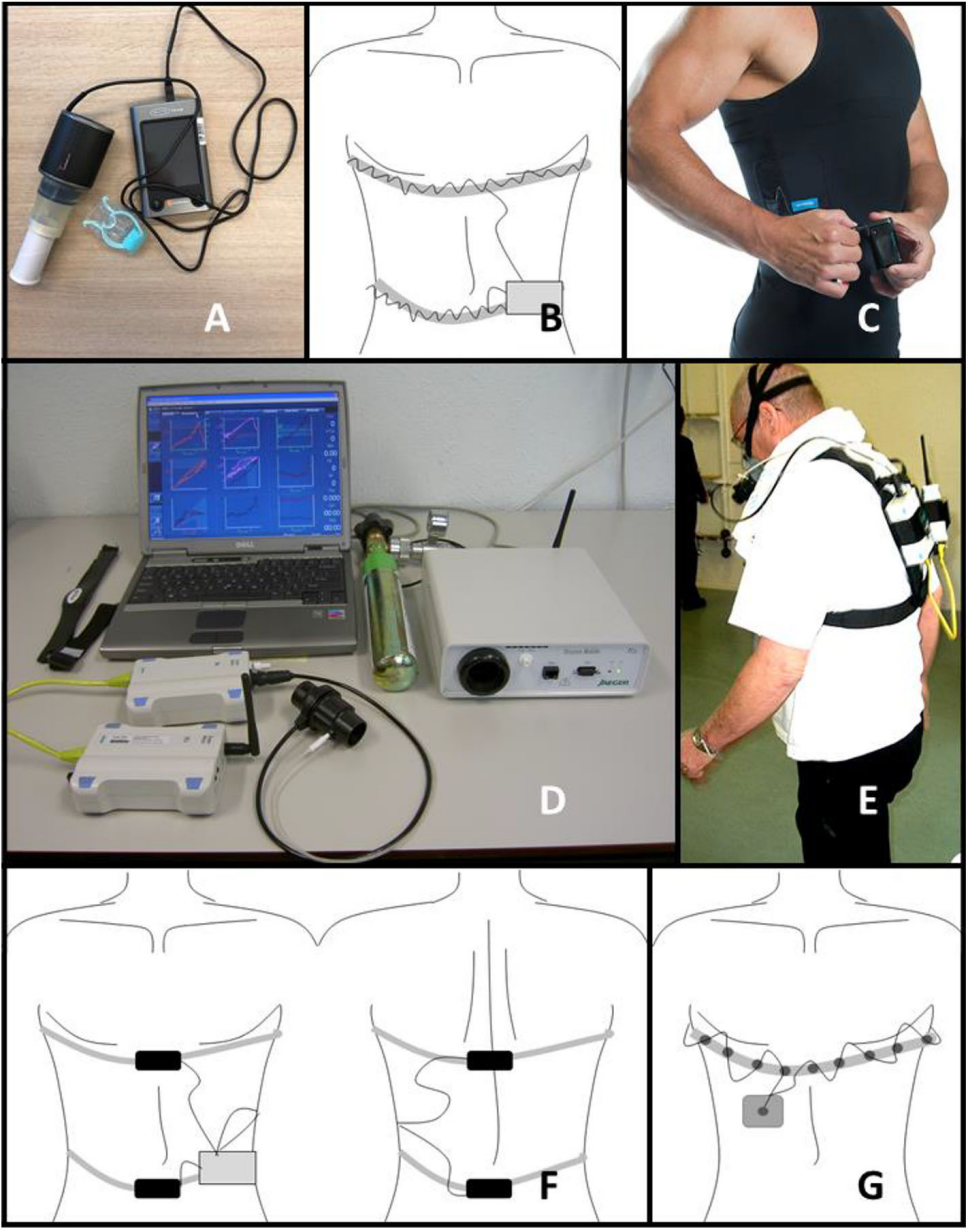
Representation of techniques. **(A)** hand-hold spirometer, **(B)** schematic overview of RIP system **(C)** RIP incorporated in smart shirt (Hexoskin) (28), **(D)** portable BbB-analyser system, **(E)** portable BbB-analyser system worn by a patient (written informed consent was obtained from the individual for the publication of this figure), **(F)** schematic overview of RMP system, **(G)** schematic overview of EIT system.

Several spirometers are available, from portable devices (26, 27) to larger less portable versions. The larger equipment is mostly used in clinic, while the portable devices can also be used at home, see Figure 2A. Some systems combine a rotating vane system to measure volume with gas analysis, the so called BbB-analyzers (29, 30) (Figures 2D,E). Wearable BbB-analyzer systems are commercially available and are mostly used for long lasting measurements (range of hours) (16, 25, 30).

Whereas, spirometry is based on measurement of flow or volume of in- and exhaled air, other methods such as respiratory inductance plethysmography (RIP) and respiratory magnetic plethysmography (RMP) measure change in chest wall volume. In 1967, Konno and Mead were the first to describe relations between the motion of the chest wall and the displacement of volume (31). Their theory assumes that the chest wall has two moving “parts,” the ribcage and abdomen (two-compartment respiratory system model). The volume displacement in the ribcage and abdomen is equal to the volume displacement at the mouth and has a linear relation with the anteroposterior movement of the ribcage and abdomen (31).

### Respiratory Inductance Plethysmography

RIP was first introduced by Cohn et al., in 1982 (32). A voltage-controlled oscillator is used to detect changes in the cross-sectional area of the torso of a subject. The assumption is that the change in cross-sectional area of both ribcage and abdomen has a (close to) linear relation to ventilation (32). The oscillator consists of an inductor and two capacitances in series (forming the tank circuit), and an amplifier in the feedback loop. The system is characterized by a central frequency or “oscillator-resonance frequency” (33, 34). The RIP techniques exist of one or two winding coil(s) around the rib cage and/or abdomen, these are the inductors of the system, see Figures 2B,C (32). A small alternating current is sent through the coils to create the inductance. The amount of inductance is not only determined by the strength of the alternating current, it is also dependent on the cross-sectional area encircled. During breathing the cross-sectional area of the coil changes, resulting in a change of inductance and a deviation from the central frequency. This deviation is determined by frequency demodulation of the system and it reflects the respiration of a subject. The output of RIP is in volts or arbitrary units (24). The main use of RIP is for the measurement of respiratory rate or dynamic volumes, and in particular TV (33, 35). Theoretically static lung volumes can be measured with RIP (34, 36, 37). The baseline of the RIP signal at rest represents rest EELV. It is important to emphasize that RIP measures chest wall volume, which is largely determined by the lung volume. However, it is also determined by the surrounding tissue. The measurements of lung volume are based on the cross-sectional area changes covered by the RIP bands. During deep breathing, the lungs will not only move in the ventral-dorsal plane. They move in the cranial-caudal plane as well. This last plane is not covered by the RIP bands, which may decrease the accuracy of this measurement technique (32, 33).

### Respiratory Magnetic Plethysmography

RMP can be used to calculate static and dynamic volumes from anteroposterior displacement of the rib cage and abdomen (24). In one of the first studies with RMP, two sensors were placed at the ventral and dorsal midline of the rib cage at nipple height and two at umbilicus height (21). As the axes of the sensors are in parallel, the sensors produce a dipolar magnetic field. The magnetic field becomes stronger when the distance between the two magnets is smaller, inducing a higher voltage in the coils. The voltage induced in the coil is inversely proportional to absolute distance between the two coils, squared. A schematic representation of the RMP system is shown in Figure 2F. The first magnetometers used in these studies contained one-axial sensors. In 1985 Levine et al. (38) introduced triaxial magnetometers. All triaxial sensors emit three orthogonal magnetic fields varying with time. Instead of only a x direction, the position in x, y, z coordinates can now be determined per sensor. In the study of Dumond et al. (39) the axial displacement of the spine and chest wall are combined with the traditionally measured anteroposterior displacements of the rib cage and abdomen. The introduction of more directions to measure ventilation increases the two degrees of freedom system to a system containing three degrees of freedom (39).

### Electrical Impedance Tomography

Another interesting potential telemonitoring technique is based on the measurement of bio impedances. In 1903 the basis of this technique was laid by Cottrell (40). This led to the development of techniques for non-invasive measurements of properties of the human body. The intra- and extracellular fluid are full of ions and electrical charged particles. The membrane between the intra- and extracellular fluid consists of non-conducting lipids, and therefore acts as a capacitance. All different tissues have a complex 3D arrangement of cells and fluids. An alternating electrical signal applied to a biological tissue, results in a complex bioelectrical impedance. This impedance is a function of the tissue composition and of the frequency of the applied alternating current (41). Electrical impedance can be measured by injecting low amplitude, low frequency alternating current through an array of surface electrodes. Other non-injecting electrodes measure the impedance. The minimum number of electrodes for measurement of impedance is two (both electrodes sent a signal and sense the signal). However, the use of at least four electrodes (two sending and two sensing electrodes) is preferred (24, 41, 42).

In the 1950s Nyboer et al. developed a method to measure blood volume changes (41), this method is called electrical impedance plethysmography (EIP) (43). Subsequently, the same research group used this method to perform pulmonary measurements with the use of two to four electrodes (44). The impedance signals measured on the chest wall, contain respiratory and cardiac components and noise. In healthy subjects, the impedance change due to the cardiac component is very small, as fluids (i.e., blood) are good electrical conductors. During breathing, the amount of air fluctuates, causing the conductivity of the lungs to change strongly. Therefore, changes in impedance can be related to changes in circumference of the chest wall (24). In EIP, changes in circumference of the thorax are only measured at the level of the electrode. EIP is mostly used for detection of respiratory rate and apnea (24).

Henderson and Webster (45) used the EIP method and developed it further into electrical impedance tomography (EIT). Multiple electrodes, usually 16 or 32, are placed around the thorax to measure the changes in conductivity during breathing (24). An image can be reconstructed of the transverse plane of the chest wall based on the multiple electrodes. This image can be used to derive (regional) dynamic and static lung volumes. The first commercially available device was produced by Dräger (40). A schematic representation of an EIT system is given in Figure 2G.

## Calibration Procedures

Calibration procedures have to be followed in order to gain high quality. Therefore, the calibration procedures and their corresponding (dis-) advantages are explained. Table 1 provides an overview of calibration time and frequency.

**Table 1:** Overview of evaluation handhold spirometers (SPIRO), breath-by-breath-analyzers (BbB), respiratory inductance plethysmography (RIP), respiratory magnetic plethysmography (RMP) and electrical impedance tomography (EIT) on calibration procedure, artifact occurrence and usability.

		**SPIRO**	**BbB**	**RIP**	**RMP**	**EIT**
Calibration procedure	Calibration time	<5 min^1^	<5 min	>5 min	>5 min	>5 min
	Calibration frequency	Always^1^	Always	Once	Once	Always
Artifact occurrence as result of	Movement	Yes	Yes	No	No	Yes
	Temperature	Yes	Yes	Yes	Yes	Yes
	Barometric pressure	Yes	Yes	No	No	No
	Electromagnetic interference	No	No	Yes	Yes	Yes
Usability	Technician assistance	No	Yes	Yes^2^	Yes^2^	No
	ADL obstruction	No	Yes	No	No	No
	Patient comfort	+	–	++	+	+

*Patient comfort; “++” very comfortable, “+” slightly comfortable, “–” uncomfortable. ^1^calibration not necessary when using an ultrasonic flow meter or factory-calibrated device, 2 only in the first measurement*.

All flow meters (except ultrasonic sensors) have to be calibrated prior to the measurement to obtain a volume derived from flow. The calibration of the flow sensor is based on a known volume (e.g., a 1 L or 3 L syringe), rather than a known flow. The ATS/ERS standard (16) recommends to calibrated clinical spirometers each day, which takes <5 min. At least once a week the calibration should be performed in different flow ranges, for which the three flow calibration is most widely used (16). In addition to the conversion from flow to volume, a correction factor is used to accurately measure volume based on the ambient temperature and pressure (ATPS/BTPS factor) (16, 25). This factor corrects for volume changes due to heating and humidifying of the air in the airways. Hereby correcting for the difference between volume of air in the lungs and same amount of air at room temperature. To correctly determine the ATPS/BTPS factor, temperature and pressure sensors should be measured accurately (16). Portable spirometer systems are often factory-calibrated (46), which means that the flow sensing unit is calibrated shortly after fabrication and is disposable after using the unit a number of times. Therefore, these systems are feasible to use at home due to the simplicity of its use. In addition to a flow calibration, the BbB-analyzer should also receive a gas (oxygen and carbon dioxide) calibration (47). The BbB-analyzer should be calibrated prior to each measurement and a technician should be available to do so (16, 25).

The calibration procedures for RIP and RMP are comparable. In all procedures, information on the chest wall properties are linked to the amount of in- and expired volume. Whereas, spirometers are mainly calibrated in a standard way (16), RIP and RMP can be calibrated in multiple ways varying in duration and complexity (48–50).

Based on laboratory tests linear systems to calibrate RIP were implemented. In these tests, the characteristics of the RIP sensors were studied (34, 51). Watson et al. (51) found that RIP sensors had a linear response to changes in circumference/cross-sectional area. Zhang et al. (33) tested a RIP system to determine if it was feasible to monitor respiration. The sensors proved to be linear in a physiological range. To gain volumes from RIP, two steps must be taken (31). First, signals from thorax and abdomen have to be summed in which the abdomen signal is multiplied with a factor K. Second, the signal has to be converted from arbitrary units or voltages to volumes with a factor M, see Equation 1.


(1) $$\begin{array}{l} {\text{Volume}}_{\text{RIP}}\;=\text{ M}*\left(\text{thorax }+\text{ abdomen}*\text{K}\right) \end{array}$$

There are two well-known manners to determine factor K:

1. Konno and Mead described the isovolume maneuver (31). In this maneuver, the subject displaces as much air as possible from the rib cage to the abdomen, without air leaving at the mouth. The linear motion of one compartment is directly related to the linear motion of the other compartment.
2. Another manner was designed by Sackner et al. (48), called the quantitative diagnostic calibration (QDC) method. During 5 to 10 min of natural breathing the factor K is derived by dividing the standard deviation of the uncalibrated signals of the abdominal and thoracic band.

To obtain factor M, two procedures can be used:

1. Spirometer-calibration (48, 52, 53): simultaneous with RIP measurement, spirometry measurements are taken. Based on comparison of the two signals, the factor M can be derived. Multiple methods can be used to calculate this factor: (multiple) linear regression, non-linear methods, neural networks and other artificial intelligence methods.
2. Fixed-volume-calibration (54–56): simultaneous with RIP measurement, the subject breathes from a bag with a known volume (e.g., 800 ml). Consequently, each breath should be approximately 800 ml. Therefore, factor M can be derived without the use of a spirometer.

The last calibration method for RIP (50, 57) to be mentioned is the least squares method, using the following equation combining factor K and M in a factor a and b for, respectively, the thoracic and abdominal band:


(2) $$\begin{array}{l} {\text{Volume}}_{\text{RIP}}\;=\text{ a}*\text{thorax }+\text{ b}*\text{abdomen} \end{array}$$

Changes in body position and/or activity are known to cause alterations in calibration factors (K, M, a and b) (58–60). Therefore, the accuracy of the measured volumes increases after calibration for each specific body position. The calibration time depends on the type of method and the number of activities and position to be calibrated and can vary between several minutes and hours. Heyde et al. (61) reported no significant variations in calibration parameters between days. Performing one clinical calibration procedure is therefore sufficient, as it can be re-used in repeated measurements.

Linear calibration systems can also be applied to RMP during quiet breathing. Levine et al. (38) were able to measure distance with an accuracy of 1 mm. They found the measurement outcomes of the sensors can considered to be linear during quiet breathing. However, during more intense breathing (during walking/cycling), non-linearities can be introduced into the measurement thereby decreasing the accuracy of the derived volumes (38). RMP is calibrated according to the same principles as RIP (21). For RMP, the first calibration procedures were also the isovolume maneuver, the QDC and LSQ method (21). However, a new method for RMP was developed; the Banzett method (49). In this method fixed calibration factors (*M* = 400 and *K* = 4) are used based on the characteristics of the magnetometers and spirometry measurements (49). In contrast to RIP, RMP can be upgraded to a three (or more) degree of freedom system (38, 39). As mentioned earlier Dumond et al. (39) introduced two extra parameters and calibrated with a multiple linear regression method. Like RIP, the calibration time of the RMP technique is dependent on the type of calibration and the number of body positions/activities to be calibrated. Calibration parameters can be determined and be re-used on another day with reliable results (60).

Impedance devices have their own unique calibration procedure. Absolute EIT is based on the exact measurement of impedance by reconstruction of an image with algorithms (14, 41). No calibration is necessary for absolute EIT. The mathematical approach to reconstruct the image from the signals of all electrodes is outside the scope of the article. The absolute EIT method creates images with many inaccuracies, due to earlier stated chest wall, cardiac and noise components (62, 63). Therefore, it is most common to follow lung impedance over time (63) as a relative value of the starting value (time-difference EIT; td-EIT). In td-EIT, the image is “calibrated” by subtracting the image of a reference point (e.g., FRC). The image change over time is therefore a result of ventilation or changes in EELV. Due to the placement of the electrodes the image is received as a circle, while the thorax has a more elliptical shape. Most systems can correct for this phenomenon, however in some equipment it can cause non-linearities. To translate impedance images to volumes, a volume calibration is in place. A (linear) relation between impedance change and TV and/or vital capacity measured with spirometry is used to calibrate the images to volumes (64). Moreover, the fixed volume calibration can also be used to derive the relation between volume and image (65). Calibration time is a few minutes, as the technician needs to determine what the baseline value is and what the linear relation between EIT image and spirometry values is (24, 66, 67).

## How Accurate are Telemonitoring Systems?

**Figure 3:**
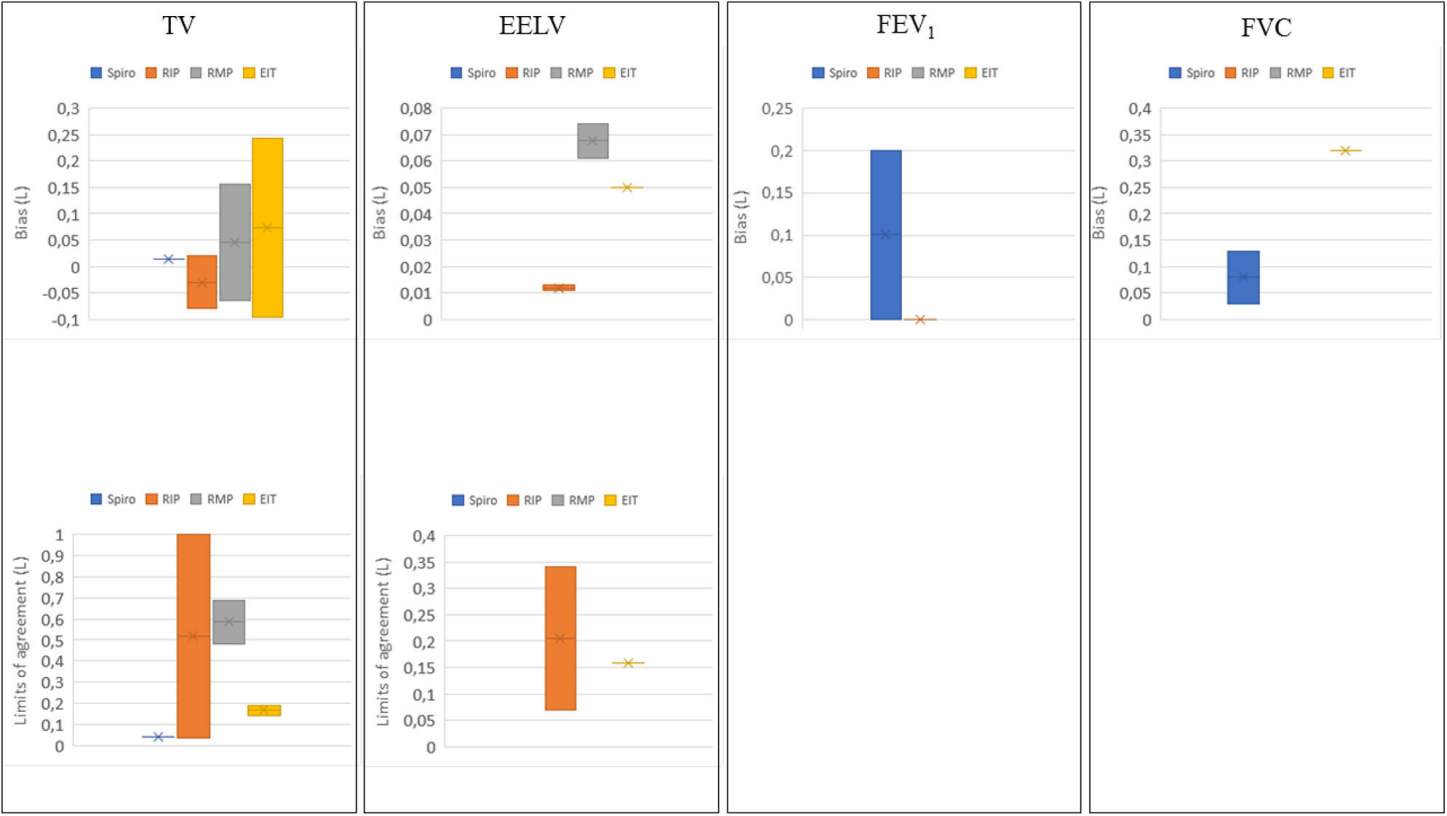
Bias and limits of agreement distribution in liters for tidal volume (TV) (37, 39, 47, 49, 52, 53, 55, 58, 64, 65, 71–90), end-expiratory lung volume (EELV) (36, 37, 87, 91), forced expiratory volume in 1s (FEV_1_) (26, 27, 64, 69, 74, 92–94) and forced vital capacity (FVC) (69, 74, 92, 93) per telemonitoring technique. Spiro; spirometry (including BbB-analyzers) in blue, RIP; respiratory inductance plethysmography in orange, RMP; respiratory magnetic plethysmography in gray, and EIT; electrical impedance tomography in yellow.

Multiple systems are commercially available (40, 68–70), and an even greater number of systems is self-made (33, 39). Not all systems have the same accuracy. To obtain good quality data in a telemonitoring setting, it is important to be aware of various techniques and their accuracy in measurement of certain parameters. An overview of the accuracy in terms of bias and limits of agreement of several lung volumes can be found in Figure 3.

### Tidal Volume

Portable spirometry systems are not used for measurement of TV. BbB analyzers are mostly validated by comparison with a computer system which mimics gas exchange of a human (47, 95). In most studies with BbB analyzers the validity of these devices is expressed by a comparison of ventilation (Ve) with the reference (68, 95). Only one study was found comparing TV measured with a BbB analyzer to a gas exchange simulator, in the study of Prieur et al. (47) a mean bias of 0.014 L was found compared to TV as simulated. However, multiple systems measure minute ventilation and respiratory rate accurately (47, 95). Therefore, we can assume that TV is also measured accurately. Since dividing the minute ventilation by the respiratory rate yields TV.

The accuracy of TV measured with RIP was investigated in several studies. In the study of Retory et al. (71) a bias of 0.040 L was found for measurement of TV, with limits of agreement of ±0.035L. Grossman et al. (55) found a bias close to zero between spirometry and RIP TV. The studies performed to investigate the accuracy of RIP to measure TV, show varying results (37, 49, 52, 53, 55, 58, 72–84). The bias is at minimum −0.080 L (77) and at maximum 0.020 L (52). Limits of agreement vary between 0.035 and 1.00 L (58, 71, 75, 85).

Only four studies (39, 49, 86, 87) are found describing the accuracy of TV measured with RMP by means of a bias. In the study of Dumond et al. (39) a bias between −0.064 L, standing in rest, and −0.502 L during running at 12 km/h, was found. McCool et al. (86) found biases of 0.038 L at rest with changes in posture, and 0.182 L during exercise. The limits of agreement were ±0.483 and ±0.687 L for, respectively, rest and exercise. Banzett et al. (49) found a 0.050 L mean difference during tidal breathing in rest. In the study of Avraam et al. (87), the bias was 0.155 and 0.144 L, for the Banzett and Sackner calibration technique, respectively.

EIT has often been used to monitor changes in lung volume uncalibrated (42). Only a few studies investigated the accuracy of volumetric measurements with EIT compared to spirometry. Balleza et al. (88, 89) Marco et al. (90) studied differences between EIT and spirometry in multiple populations and found biases for TV between −0.003 L and 0.218 L. Ngo et al. (64) found a bias of 0.243 L for TV. Sosio et al. (65) found a bias of −0.095L. Limits of agreement in these studies vary between ±0.188 and ±0.145 L.

In conclusion, BbB-analyzers can be considered accurate in the measurement of TV. Moreover, accurate qualitative values of TV can be obtained from RIP and to a lesser extent from RMP and EIT. The bias for TV measurement between RIP and spirometry is low, which implies that the technique on average is very accurate. In RMP and EIT, more variability has been found in the bias. The variation can be explained by the use of different calibration techniques, the number of RMP-sensors or EIT-electrodes and the appearance of artifacts. Moreover, it should be kept in mind that volumes measured with RIP, RMP and EIT techniques will never agree perfectly with spirometry, as the measurement mechanism is different.

### Functional Volumes

Spirometry is stated as the gold standard for measurement of FVC and FEV1 (16). Portable spirometer systems are mostly validated based on non-portable systems. The Escort handhold Fleish pneumotachograph showed a mean (SD) difference for the FEV1 of − 0.050 (0.150) liters, for FVC 0.030 (0.280) liters (26). Likewise, most handheld spirometers were reported to underestimate the FEV1. Limits of agreement vary between 0.030 and 0.440 L for FEV1 and between 0.050 and 0.560L for FVC (26, 92, 93, 96). The accuracy of measurement of FEV1, based on the bias compared to clinical spirometry is at minimum 0.001 L (94) and at maximum 0.200 L (27). For (F)VC, the bias varies between 0.030 L (26) and 0.130 L (69). BbB-analyzers are not used for measurement of FVC and FEV1. In conclusion, not all portable devices are fit for these measurements, the bias between different devices and a clinical spirometer can be up to 0.20 L. It is therefore very important to select the most appropriate (mobile) device.

Functional volumes can also be measured with RIP technique. In the study of Tobin et al. (74) FEV1 was measured with both RIP and spirometry. A maximum deviation from spirometry values was found of 0.180 L (10% on an FEV1 of 1.800 L), the minimum error was close to zero. The difference between spirometry and RIP is explained by Tobin et al. (74). They stated that the difference between both techniques is likely to result from the assumption that chest wall has two degrees of freedom in motion while at least three degrees of freedom during more demanding situations are required. Moreover, the difference in operation principle (flow vs. chest wall movement) is also used to explain the low accuracy. However, they stated that it would be possible to perform categorization of breathing patterns (74). They hypothesize that RIP could be used to detect decline of lung function quantitatively or percentual relative to a starting value.

No studies were found including the measurement of functional volumes such as FEV1, and (F)VC with RMP. One study was found to evaluate the measurement of FVC with EIT. Ngo et al. (64) found a bias of 0.320 L for FVC with limits of agreement of ±0.370 L. This implies that EIT seems to be not accurate enough for functional lung volume measurement.

### End-Expiratory Lung Volume

In theory, all evaluated techniques, except spirometry, can be used to assess static lung volumes directly (19, 36, 87, 91). In spirometry, an indirect measurement can be used to obtain static volumes, however this is outside the scope of the article (97). The accuracy of RIP to measure static volumes was determined in two studies. Neumann et al. (36) compared changes in end-expiratory lung volume (EELV) measured with the multiple breath nitrogen washout technique with those measured with RIP in adults and found a mean deviation of 0.0116 L (SD: 174.1). Leino et al. (37) found a mean difference in ΔEELV of 0.013 L (SD: 35), comparing spirometry and RIP.

In the study of Avraam et al. (87) subjects had to induce ΔEELV during measurement with RMP and spirometry. Avraam et al. found a bias between the outcomes of both methods of 0.061 and 0.074 L for the two calibration techniques. For the RMP technique no limits of agreement are presented in the articles. In the study of Grivans et al. (91) changes in EELV as a result of positive end expiratory pressure are measured with spirometry and EIT. The bias is 0.050 L in patients, with limits of agreement ±0.159 L (91).

In conclusion, on average, all three techniques can be used to assess EELV accurately. The limits of agreement in EELV measurement suggest that the agreement between spirometry and EIT/RIP is variable. This could be explained by the fact that in two (37, 91) of these studies, spirometry was used as comparison method. Although it is known that spirometry is not the optimal method to measure EELV. Only changes over time can be tracked with spirometry but an absolute lung volume cannot be measured. Opto-electronics, gas dilution or body plethysmography would be the preferred method to measure EELV. A last discussion point is that all studies measured EELV during mechanical ventilation by PEEP induced changes in lung volume. Therefore, the accuracy of absolute volumes measured in awake subjects remains unknown, also during movement and changes in position of the subject.

## Factors Affecting Data Quality

Artifacts can drastically decrease the quality of the measured data, so insight under which conditions they occur can make it easier to choose an appropriate monitoring system. Below we will discuss three common causes of inaccuracy; movement, temperature/barometric pressure and electromagnetic interference. See for an overview of occurrence of artifacts as a result of these factors (Table 1).

### Movement

Wearable spirometry systems can be affected by movement (98). Especially at higher intensities, leakage due to a poor fit of the mask or mouth piece introduces deviations from the true volumes (16). *In vitro* studies show that movement of a subject causes inaccuracy of all measured volumes (39, 60, 99). In RIP and RMP the inaccuracy due to movement is mainly caused by slight displacements of the sensors on the chest wall. Moreover, in RMP sensors can become non-linear at higher intensities resulting in outcomes outside the calibrated range (38). In the studies mentioned earlier, the bias and limits of agreement increase with increased activity (39, 52, 55). However, during exercise measurement of dynamic volume with RIP are still comparable to spirometry values. The inaccuracies in these techniques can be decreased by filtering of movement artifacts (100). In theory, changing posture could affect the baseline of the signals of RIP, RMP and EIT, which affect EELV measurement. The baseline shift is a result of a changing circumference due to posture change, which could be mistaken for changes in lung volume. In contrast to RIP, the change in posture does not affect the measured circumference but it could change the impedance due to a shift of organs.

### Temperature and Barometric Pressure

Most spirometry systems work with an ATPS/BTPS correction based on the temperature and barometric pressure measured prior to the measurement (16, 25). Therefore, the accuracy of the correction can be affected by changes in temperature and barometric conditions during the measurement (16). It is common knowledge that resistance or impedance of a system is dependent on ambient temperature. In theory, temperature changes can cause signal drift in RIP/RMP and EIT if not compensated (34, 101). Watson et al. (51) evaluated the stability of baseline of the Respitrace RIP system over a period of 12 h, in which no significant baseline drift was found. In their study the RIP system contained a module for thermal compensation. Mannée et al. determined a digital correction for temperature changes (34). RMP sensors can be sensitive to temperature changes, it is known that all magnetic material experience a change in magnetic flux when temperature changes (102). In some studies, EIT is used to monitor temperature (103). It is obvious that temperature influences can be prevented by analog or digital temperature correction.

During home monitoring, patients should be able to perform their normal daily activities, including walking from one room into another room or leaving the house. In this case, temperature (and barometric pressure) of the environment could change. In spirometry measurements, changes in temperature will affect the accuracy of the lung volumes measured. Most devices only determine the ATPS/BTPS correction factor prior to the measurement (68). The measurement of functional volumes is (1) short and (2) performed in an environment without changing conditions. Therefore, functional measurements are likely to be accurate. However, in longer measurements of TV with a BbB-analyzer, temperature or barometric pressure changes will induce inaccuracies in TV as the ATPS/BTPS correction factor will not be continuously updated. In RIP, RMP, and EIT, temperature changes will especially affect the measurement of absolute volumes, and most likely result in baseline drift. *In vitro* studies can be used to determine the temperature dependency of the devices and based on this knowledge corrections can be performed.

### Electromagnetic Interference

Another factor which can induce artifacts is electromagnetic interference. In RIP, the inductance created in one band can disturb the inductance of the other band, this is called magnetic coupling (33). Moreover, moving metal objects or mobile devices in a close range (<15 cm) toward the bands can disturb the inductance (99). In RMP, it is possible that the magnetic field of the sensor is disturbed by surrounding electrical systems creating their own magnetic field. Electromagnetic interference (from e.g., mobile devices or low frequency interference) can affect the signals of the EIT system (104).

In most cases, this problem is easily solved by taking away any objects which could possibly interfere or correcting for the electromagnetic interference of the object. In EIT, background noise from other e.g., machines or measurement devices, can easily be removed from the EIT signals in two manners, by using frequency bands to filter noise or by using the td-calibration (41). In td-calibration background noise should not change during a measurement, since this affects accuracy.

## Usability and Comfort

One of the factors determining how feasible a device is for home monitoring, is the necessity of a technician to assist the procedure. Moreover, the usability of a technique in telemonitoring is based on the comfort of a patient; as this results in low or high willingness of the patient to cooperate. A method should not interfere with tasks of daily living. Whenever a patient is not comfortable with the use of a certain device, this could result in missing data or data with low quality. See for an overview of usability in terms of technician assistance, obstruction of activities of daily life (ADL) and patient comfort (Table 1).

Spirometers are used for short-lasting maximal lung function measurements (FEV1, (F)VC) (16), while BbB-analyzers can be used to measure TV and ventilation continuously. Technician assistance at home is not necessary with functional measurements (105). Instruction for breathing maneuvers can be given by a video or written step-by-step plan (105). A technician is necessary in BbB-analyzer measurements to first apply all equipment and then calibrate the equipment (30). It can be hypothesized that a daily measurement of functional lung volumes is not burdensome to patients. However, if TV during certain tasks or activities are measured with BbB-analyzers, the burden will increase. During these measurements the patients must wear a full portable system with mask (30) and will therefore be restricted in their normal activities.

RIP and RMP are used to assess TV and respiratory rate (32, 39, 58). Technician assistance is necessary for the calibration procedure of both RIP and RMP, however due to the reusability of the calibration a technician should be available only once (61). In most studies, researchers apply the thoracic and abdominal band of RIP on the subject (50, 58). However, theoretically patients could be instructed to do this themselves. Especially with a shirt with incorporated RIP, such as the LifeShirt (80) or Hexoskin (see Figure 2C) (106), this would be a very easy task. In contrast to measurement with a BbB-analyzer, RIP is hypothesized to be very comfortable to wear. The straps (or shirt with straps) are (1) easily worn below own clothes, (2) skin-tight, and (3) without any protrusions that could make certain positions non-comfortable and therefore, RIP does not interfere with daily activities. In contrast to RIP, RMP is more difficult to apply, subjects would not be able to place the dorsal sensors by themselves. RMP should be applied by technicians. To the knowledge of the authors, the comfort of patients during RMP measurements has never been evaluated. RMP is hypothesized to be slightly less comfortable to wear, compared to RIP. The magnetometers can be (1) easily worn below own clothes. However, the sensors are not flexible like RIP, and therefore certain positions could be uncomfortable. The measurements should not interfere with ADL.

EIT is used to assess (changes in) EELV and ventilation (41). Technician assistance is necessary for the calibration procedure. EIT systems work with multiple electrodes, which mostly all have to be applied separately. Only a few systems have electrodes incorporated in a strap (65). An EIT system without strap would be difficult to apply by a patient, and therefore technicians should support. EIT can be easily worn below own clothes, and without any protrusions that could make certain positions non-comfortable. Therefore, EIT does not interfere with daily activities. Compared to RIP, only one thing makes this technique slightly less comfortable, electrodes must be taped to the skin (except for the EIT strap systems). The authors did not find any information on the replicability of a volume calibration, e.g., after EIT had been removed and reapplied or after the starting point had been changed. Therefore, it would be necessary to have a technician present to determine the starting point in each measurement.

## Potential Applications

The best measurement method for TV measurement should be chosen based on the length of the measurement the number of measurements, and the activities during the measurement. First, the longer the measurement the more appropriate it would be to choose for RIP. Based on the limits of agreement found in studies measuring TV with RIP (58, 71, 75, 85), the accuracy of RIP is likely to increase with longer measurement. Therefore, it is suggested that RIP is used in measurements with a duration of multiple minutes to hours or days. A BbB-analyzer would be the method of choice for shorter measurements. Second, for a one-time measurement of TV it is best to use a BbB-analyzer. Although it is less comfortable than RIP, it is less time consuming to use the BbB-analyzer in a one-time measurement, as the calibration procedure of RIP is longer. Third, the activities during the measurement should be considered. In measurements with multiple activities and positions, a longer calibration procedure is necessary for RIP and BbB-analyzer could be more appropriate. However, if activities involve changes in environmental conditions (temperature, barometric), RIP would be suitable.

Handhold spirometers can be used best to measure functional volumes. However, in a combination of tidal volumes and functional measurement, RMP could be considered. Although RMP has never been studied during the measurement of functional volumes, it has potential. During the measurement of functional volumes, there are more chest wall movements than only in the two areas measured with RIP (two degrees of freedom) (74). RMP can be used to also consider upward movements (i.e., distance between abdominal and thoracic sensors; three degrees of freedom).

RIP, RMP, and EIT might all be appropriate to measure EELV. It should be considered that during position changes baseline values might change. To the knowledge of the authors it has never been investigated if changes in baseline impedance (EIT) or voltage (RIP/RMP) are comparable to the physiological changes in EELV during these position changes. One of the advantages of EIT above RIP and RMP is that it is possible to localize a change in EELV to a region of the lung below the EIT sensors.

## Future Developments

Some suggestions are made to increase data quality and study design in the future. First, the use of EIT in a home setting should be explored. It could be an interesting method to visualize lung dynamics at a distance. Second, to increase data quality the occurrence of artifacts and the removal of these artifacts should be studied. Third, the measurement of EELV with telemonitoring techniques has not been explored extensively. EELV is an important volume in for example chronic obstructive pulmonary disease (107, 108). Monitoring these volumes at home could possibly increase the effectiveness of telemonitoring systems (109).

## Summary

In this review, we described the operation principle of portable spirometry/BbB-analyzers, RIP, RMP and EIT. All methods were assessed on calibration time, accuracy of measurement of static and dynamic lung volumes, occurrence of artifacts (e.g., distortion by movement or pressure and temperature changes), and technician' and patient' effort in the measurement. In future work, this review can be used to select the method of preference by listing the various requirements in order of importance and selecting the best fitting technique.

## Author Contributions

DM, FJ, and HH wrote and edited the manuscript. All authors contributed to the article and approved the final submitted version.

## Conflict of Interest

HH receive finanacial support during the writing of this review from Chiesi in the form of an educational grant. The remaining authors declare that the research was conducted in the absence of any commercial or financial relationships that could be construed as a potential conflict of interest.
